# The Mexican Dysentery Remedy Again

**Published:** 1895-06

**Authors:** R. T. Knox

**Affiliations:** Gonzales


### Correspondence.

### The Mexican Dysentery Remedy Again.

LETTER FROM DR. KNOX.

GONZALES, May 18, 1895.

*Editors Texas Medical Journal:*

At the request and urgent wish of many correspondents to learn more of it, I again write of the merits of the *Chapparro*

*Armagosa*, an unpretentious, ugly, thorny, forbidding shrub, abundantly growing in Southwest Texas, its uses in dysentery, etc., a condition of bowels so uncontrollable by our best physicians, and so acknowledged by one and all.

Some three years since I wrote up my own case and its treatment for the benefit of all like sufferers. This for our State Medical Association of Texas, where, at the meeting at Tyler, if read at all, was no doubt read by caption and referred to the publishing committee. These learned gentlemen deeming it too previous, or one case as not sufficient to establish its claim, "hove" it over among the rubbish; let it slide; urging "that the paper should be boiled down," I presume, reduced in volume, while only a short time before much space was allowed or given to the claim of *arsenite cupri* in like condition.

Many months after this time this paper was published in the TEXAS MEDICAL JOURNAL, and attracted marked attention, it being read wherever medical literature was read, and treasured by many hundreds of sufferers; so much so, letters of inquiry and application came to me from all points. In every State it was sent to different parties and used by them, many claiming and calling it the "Elixir of Life," reboiling their supplies and using it until more reached them. A pharmacy firm readily embraced it, making a fluid extract of it; now since have it in tablet form. It has been registered and *has come to stay*, as many other vegetable products have done before, as nux vomica and its alkaloids, ergot, ipecac, cinchona, and hundreds of others, and more recently spartine, and others of its like. Now, how have these, severally and all, been established as reputable drugs and procured from the vegetable kingdom? And as yet the field not explored but many more things to hear from having equal merit with any we now know of. Go with me back into the fifties, see just how procured for use then—the now much used *viburnum pruni foliatum*. The bark was obtained, a decoction made and preserved, and successfully so.

I do not wish to enlarge on my former statement of this drug, but it has further benefits; it is anti-malarial largely, its bitter principal may be its possible good. But recently my attention has been called to its use by enema, in chronic dysentery, by my friend Dr. Lankford, of San Antonio. I have so used it, and find it works well; then, if there be any hemorrhoids, it will surely relieve them greatly.

I beg to suggest that more benefit arises from a decoction or strong tea, and this drank often and freely, rather than with any alcoholic compound, as alcohol is not admissible in any bowel trouble when there is ulceration. I think, if dispensed by our pharmacists, better ground dry and in packages as the Quaker herbs, and used as needed.

Hoping my further promulgation of this simple drug may be the means of much good to all afflicted thus, as I feel it has already been to many, myself as well, I take pleasure in further arguing this matter. I am yours truly,

R. T. KNOX, M. D.

